# Does living with children or financial adequacy mitigate the impact of IADL limitations on older adults’ well-being? Findings from the Longitudinal Indonesian Family Life Survey

**DOI:** 10.1186/s12877-025-06634-w

**Published:** 2025-12-27

**Authors:** Farma Mangunsong, Tawanchai Jirapramukpitak, Sureeporn Punpuing, Sutthida Chuanwan, Dyah Anantalia Widyastari

**Affiliations:** Institute for Population and Social Research (IPSR), Mahidol University, Nakhon Pathom, Thailand

**Keywords:** Aging, Depression, Happiness, Household, Ordered logistic regression, Self-rated health

## Abstract

**Background:**

Instrumental activities of daily living (IADL) limitations reduce the well-being of older adults. However, it remains unclear whether co-residence with children or other family members provides sufficient support to mitigate this impact. This study examined the longitudinal effect of IADL limitations on well-being and assessed whether the presence of children or other sociodemographic characteristics moderated the relationship.

**Methods:**

821 participants from waves 4 and 5 of the Indonesian Family Life Survey were analyzed. IADL limitations were measured by the presence of difficulties in shopping, preparing meals, and taking medicine. Well-being was assessed by a composite index combining happiness, self-rated health, and depressive symptoms. Living arrangements were classified based on household composition. An asset index was built by applying tetrachoric principal component analysis (PCA). Ordered logistic regression models were used.

**Results:**

IADL limitations in wave 4 (AOR 0.41, p-value 0.003, 95% CI 0.23 0.75) and worsening IADL limitations over time (AOR 0.50, p-value 0.000, 95% CI 0.36 0.70) were independently associated with poorer well-being in wave 5. Higher levels of household assets (AOR 1.67, p-value 0.003, 95% CI 1.19 2.34) were significantly associated with good well-being. The decrease in assets over time (AOR 0.62, p-value 0.003, 95% CI 0.45 0.85) was independently associated with poorer well-being. No significant interaction effect was found between IADL limitations and living with adult children (OR 0.41, p-value 0.014, 95% CI 0.20 0.84) or other household members.

**Conclusions:**

Co-residence with children or other family members does not appear to mitigate the adverse effects of IADL limitations on the well-being of older adults. Instead, policies aimed at strengthening financial adequacy and maintaining functional capacity could be more effective in enhancing their well-being.

## Background

Well-being has become an increasingly important concept for understanding an individual’s overall welfare. Commonly associated terms include quality of life (QoL), life satisfaction, happiness, depression, and self-rated health. These terms are often used interchangeably in discussions of well-being, although they reflect different aspects. QoL generally encompasses multiple life domains—such as income, education, and health—and can serve as a broad indicator of overall life satisfaction [1]. Some studies, however, focus specifically on life satisfaction [2] or self-rated health [3]. Other research highlights emotional dimensions, using happiness to reflect feelings of pleasure [4] and depression to capture experiences of psychological distress [5].

A decline in well-being commonly accompanies advancing age, with deteriorating health being a major contributing factor, as highlighted by the World Health Organization [6]. For example, in 2021, health satisfaction scores in Indonesia revealed a noticeable decline with age [7]. On a scale of 100, individuals aged 24 or younger had an average score of 80.87. This decreased to 79.71 for those aged 25–40, 75.70 for individuals aged 41–64, and 69.45 for those aged 65 and older. Similarly, personal life satisfaction scores, as a representative of well-being, decreased with age. The scores were 71.92, 72.39, 71.42, and 69.47 across the same age groups.

Concerns about declining health and life satisfaction have become increasingly salient alongside the growing proportion of older adults in Indonesia. The percentage of older adults, individuals 60 years old or above, as defined by the Government of Indonesia [8], increased from 7.59% of the total population in 2010 to 12.00% in 2024 [9, 10]. Meanwhile, the old dependency ratio increased from 11.95 in 2010 [9] to 17.08 in 2023 [11]. Regarding living arrangements, in 2015, the percentages of older adults living alone, with a spouse, with immediate family (children and a spouse), in a three-generation household, and with others were 8.90%, 19.96%, 26.84%, 35.62%, and 8.66%, respectively. In comparison, the corresponding figures in 2010 were 7.10%, 22.07%, 33.66%, 34.68%, and 2.50%. These trends highlight an overall increase in multigenerational living—particularly with immediate family and in three-generation households—suggesting a growing caregiving burden on younger household members.

Well-being is essential as it reflects an individual’s capacity to manage emotions, engage in social and independent activities, and reduce the risk of self-harm [12]. It is also a reflection of an individual’s feeling of happiness or hedonic aspect, satisfaction toward life domain or evaluative aspect, and self-awareness of life purpose or eudaimonic aspect [13]. Older people with good well-being, such as those who are happier, tend to experience slower declines in physical function [14] due to positive self-perceptions, a healthy self-concept, and active social connections with family and community [15]. Thus, physical function reflects not only physical health, but also individual social function.

According to self-determination theory, health influences well-being by affecting an individual’s competence, autonomy, and relationships with others [16]. For instance, older adults with functional limitations in IADL, which may signal early cognitive decline [17] or the presence of multimorbidity [18], often report poorer self-rated health [18], an increased risk of depression [19, 20], and reduced happiness [21]. These outcomes are linked to fewer opportunities for social activities and solitary hobbies that contribute to happiness [22]. Struggles in managing daily tasks and reliance on others for basic care can undermine feelings of competence and autonomy [23], contributing to diminished self-worth and increased depression [24, 25]. At the same time, frequent interactions with caregivers and family members may strengthen their sense of relatedness due to the daily support they receive. Thus, having IADL limitations is not only about physical decline, but also linked to a cognitive decline and a less social role [26, 27]. In other words, having IADL limitations is a sign of having functional declines.

Living arrangements play a significant role in providing social support to older individuals, aligning with the person-environment fit theory [28]. In Indonesia, many older adults live with their adult children due to the need for physical [29], financial [30, 31], and emotional support [32]. Meanwhile, the others live alone without children under challenging health and financial circumstances, relying on community-based support and small remittances from their children [33]. Conversely, some older adults in Indonesia live alone or with only a spouse because they have no IADL difficulties and can maintain physical independence [34]. In other Southeast Asian countries, such as Singapore and Thailand, living alone implies receiving daily physical and mental support from the immediate social circle [35, 36] as well as having sufficient financial means [37].

Living arrangements can serve as a form of social support that contributes to older persons’ well-being, as suggested by social support theory [38]. Co-residence with adult children is often associated with filial piety from children to parents in Asian cultures [39, 40], including Indonesia [41]. However, the impact on well-being varies. Living with adult children, together with a spouse, may improve older adults’ mental health due to physical and financial support from adult children [42]. Having financial adequacy and functional independence also contributes to the well-being of older adults living with children [43, 44]. Conversely, it can also lead to depression if adult children are neglectful [45]. Meanwhile, some older Indonesians living apart from their children report higher happiness when they have a stable financial and physical state [32, 46].

**Fig. 1:**
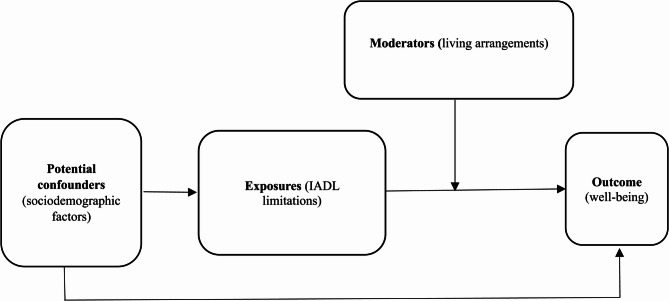
Research’s framework

The theoretical foundation and empirical findings above underpin the research framework presented in Fig. 1, adapted from a causal diagram known as a directed acyclic graph (DAG) [47]. The theories emphasize the detrimental impact of functional limitations on well-being and examine the potential moderating role of living arrangements. Accordingly, this research identifies IADL limitations as exposures, living arrangements as potential moderators, and well-being as the outcome. Sociodemographic factors, including age, gender, educational attainment, and economic status, are considered potential confounders, as they influence both the functional decline and well-being. For instance, older age, males, higher educational attainment, and stable economic status are generally associated with better well-being. Older adults may have more leisure time [48]; men often bear fewer domestic responsibilities within households [2]; higher education is associated with broader social networks [42]; and stable economic status enables individuals to meet daily living expenses [49]. While advancing age is typically associated with functional decline, being male, well-educated, and economically stable are often associated with better functional capacity, partly due to greater access to nutritious food and quality healthcare [6].

Previous research in the aging society has examined the links between functional limitations and well-being, as well as between functional limitations and living arrangements, and between living arrangements and well-being. However, a gap remains in understanding whether living arrangements moderate the relationship between functional limitations and well-being. This study aims to address that gap by examining whether co-residing with adult children or other household members contributes to better well-being among older adults with functional limitations. Hence, this current study hypothesizes that: (1) older adults with IADL limitations and worsening limitations over time will have poorer well-being, and (2) these associations are influenced by the presence of household members, particularly children. Furthermore, we explore whether other individual characteristics moderate these associations.

## Methods

### Data

Data for this study were sourced from the Indonesian Family Life Survey (IFLS) administered by RAND [50]. This interviewer-administered survey covers 13 of 27 provinces in Indonesia and encompasses five waves: wave 1 in 1993/1994, wave 2 in 1997, wave 3 in 2000, wave 4 in 2007/2008, and wave 5 in 2014/2015. The survey used paper-based questionnaires from wave 1 through wave 4, before shifting to the Computer Assisted Personal Interviewing (CAPI) system in wave 5. The IFLS tracked the same individuals and households over time, including those that branched off from the original households. Over 90% of the 7,224 households in wave 1 were successfully recontacted in subsequent waves, including those where the members had passed away. Almost 87% of the households in wave 1 participated in every subsequent wave. The survey covered a wide range of topics, including sociodemographic and health-related information. The high retention rate and the survey’s rich information render the IFLS a valuable resource for longitudinal behavioral studies [51].

### Study design and sampling

**Fig. 2:**
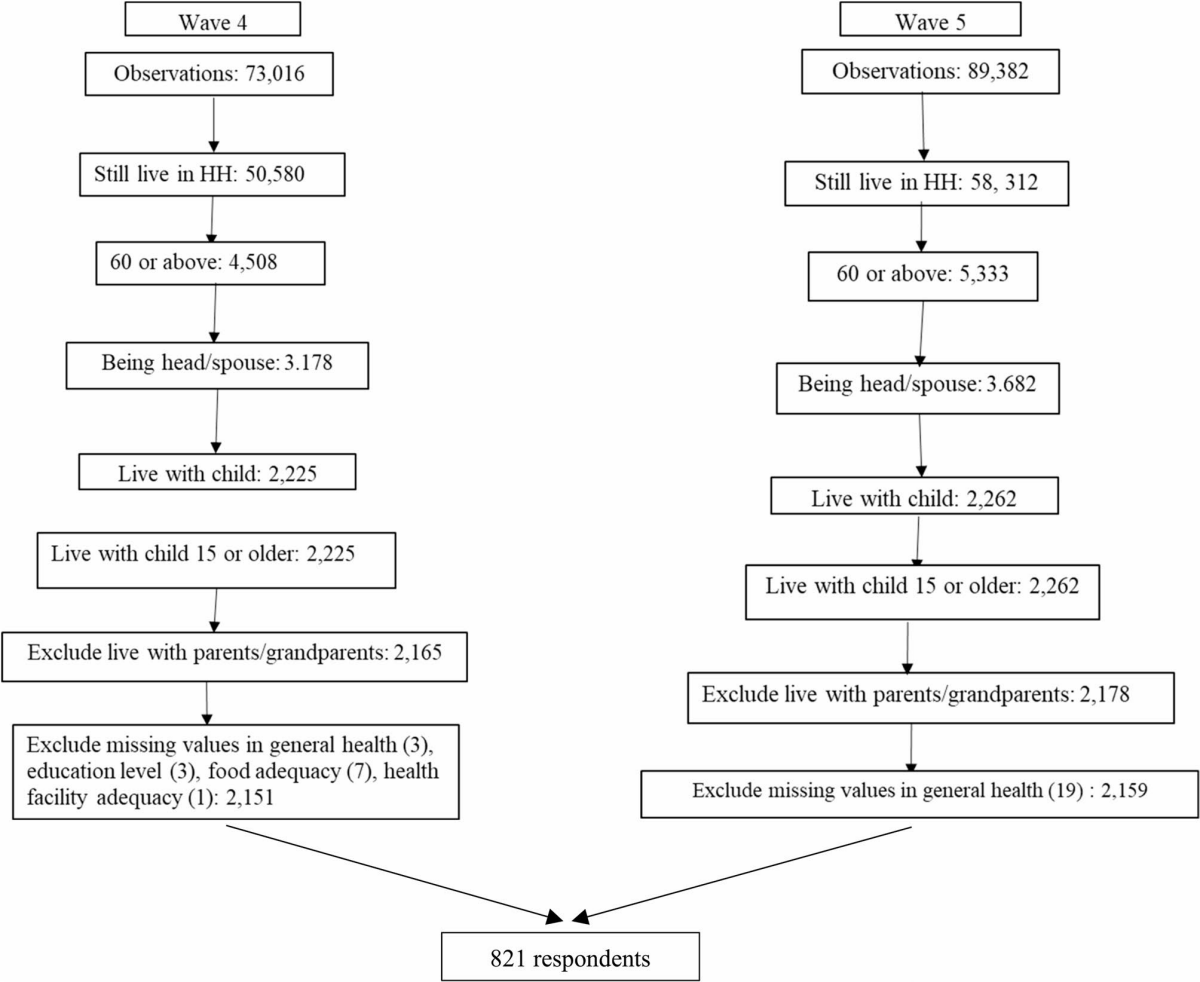
The sampling flow chart

The IFLS wave 4 is the baseline, and wave 5 is the follow-up. Using both waves is a benefit for this panel longitudinal study, as two components of well-being in this study-general happiness and 10 depressive symptoms-were introduced in wave 4 and remain available in wave 5. The criteria for participants included in this study were being 60 or older in wave 4, being a head of household or the spouse in both waves, and not living with parents, parents-in-law, or grandparents to ensure that the observations were not caregivers [29], and living in the same household in both waves. The criterion of being the head of household is required to identify the relationships between household members in the roster and the head of household, and construct the living arrangements variable. Participants were retained if they responded to whether they lived with a child or were childless. A small number of participants with missing values for educational attainment, depressive symptoms, perceived self-adequacy of food consumption and health care in wave 4, and self-rated health in waves 4 and 5 were excluded. However, self-adequacy of food consumption and health care were excluded from the analysis, as they reflect socioeconomic status, such as financial status [52]. The sampling flow chart is presented in Fig. 2.

Based on the criteria, there were 2,151 in wave 4 and 2,159 observations in wave 5. Of those observed in wave 4, 1,330 individuals were not present in wave 5. They consisted of 489 deceased individuals, 60 individuals who moved to different households, and 781 observations who could not be found in wave 5. Thus, 821 observations in wave 4 matched those in wave 5.

### Outcome

The outcome was well-being in wave 5. To capture a more comprehensive measure of well-being, three components were included: general happiness, self-rated health, and depression, representing hedonic, evaluative, and eudaimonic aspects, respectively. The general happiness was adapted from the United States General Social Survey; self-rated health was drawn from the Health and Retirement Study (HRS); and depression was assessed by depressive symptoms based on a short version of the Center for Epidemiological Studies of Depression Short Form (CES-D-10) [53]. The first component of PCA was applied to build a valid well-being index [54]. The polychoric PCA was used for the mixed measurement levels of the binomial variable for depression and the ordinal variable for the others. Following the Kaiser criterion, the first component was valid as its eigenvalue exceeded 1 [55]. The eigenvalue was 1.41. The indices were then grouped into quartiles to ensure adequate distribution of observations across subgroups.

This study assessed general happiness and self-rated health using a 4-point Likert scale and gauged depression with a binary option. In the IFLS questionnaire, the question of general happiness was “Taken all things together, how would you say things are these days?” in four categories: very unhappy, unhappy, happy, and very happy. The self-rated health question was “In general, how is your health?” with options of unhealthy, somewhat unhealthy, somewhat healthy, or very healthy. These ratings were coded on a scale from 1 to 4, with higher values indicating greater happiness and better self-rated health.

We made a few modifications and conducted a reliability test on the 10 depressive symptoms. The question of each symptom was “How do you feel in the past week?” The symptoms were feeling bothered, difficulty concentrating, depressed, full of effort, hopeful about the future, fear, restless sleep, happy, lonely, and not keeping going in the last week. The questionnaire coded the occurrence of each item from 1 to 4, meaning rarely or never, some days, occasionally, and most of the time. Since being hopeful about the future and happy were positive statements, we reversed the scales to align with the negative mental health statements [56]. Depression was determined by a total median value of each symptom as a cut-off point [56]. The cut-off point for depression was 12. A score above 12 indicated depression and was coded as 0. Being at or below 12 indicated no depression and was coded as 1. As a rule of thumb, a Cronbach’s alpha greater than 0.5 is acceptable to represent a reliability [57]. The Cronbach’s alpha was 0.71, indicating that the 10 items were reliable in representing depression.

### Exposure

The exposure was IADL limitations and the changes. IADL limitations usually come first among older adults, as found in China [58]. Having IADL limitations is more common than activities of daily living (ADL) limitations among older adults, as found in Poland [59] and Nepal [60]. The same IADL items from waves 4 and 5 were retained to ensure consistency between the two waves. They were to shop for personal needs, to prepare meals, and to take medicine. In wave 4, these tasks were originally grouped into three categories: easily, with difficulty, and unable to do [53]. In wave 5, they were grouped into four categories: easily, with difficulty, can’t do without help, and unable to do [51]. For this study, IADL limitations were categorized into having no IADL limitations (for individuals who can perform all the tasks easily) and having any IADL limitations (for those facing any difficulties) [61, 62]. Since IADL limitation is a time-variant variable, the changes from wave 4 to wave 5 were grouped into two categories: (1) same status (either having no or having any IADL limitations in both waves) or experiencing an improvement from having any in wave 4 to having no IADL limitations in wave 5; (2) worsening IADL limitations over time (from having no in wave 4 to having any IADL limitations in wave 5).

### Confounders

The confounders were selected based on the conceptual framework and evidence in previous studies of functional limitations and well-being. They were age, gender, educational attainment [18], economic status represented by a household asset index [61], and well-being in wave 4 to capture the time-invariant effect on well-being in the consecutive wave [63]. Age was categorized into 60–69 and 70 or above. Educational attainment was categorized in ascending order into never in school or not completed elementary school, elementary school, junior high school, senior high school, and diploma/university. Gender was categorized as male or female.

By applying the first component of the PCA with tetrachoric, the asset index in tertiles was constructed based on yes/no binomial answers for household ownership of each asset [64]. The assets encompassed house and land, another house, other land, poultry, livestock, plants, vehicles, appliances, savings, accounts receivable, jewelry, and other assets. The 1 st, 2nd, and 3rd tertile refer to low, middle, and high economic status, respectively. Since the asset index is a time-variant variable, the changes were ordered into decreased, same, and increased assets.

A few modifications were also made to construct a well-being index in wave 4. The scales for each item of depressive symptoms ranged from 0 to 4, representing never, rarely, some days, occasionally, and most of the time, respectively. The scales of hope about the future and happiness were reversed first to align with the negative mental health statements. Then, 0 was included in 1 to align with wave 5. The cut-off point for depression was set at 10. A score above 10 indicated depression and was coded as 0, whereas a score of 10 or lower indicated no depression and was coded as 1. The Cronbach’s alpha was 0.53, indicating fewer depressive symptoms perceived in wave 4 or younger old age than in wave 5, and the eigenvalue was 1.37.

Some covariates were recategorized for exploratory analysis purposes. Educational attainment was grouped into: (1) never in school or not completed elementary school, and (2) elementary school or higher. The asset index was grouped into: (1) low, and (2) middle and high. Asset changes were categorized as follows: (1) the same and increased assets, and (2) decreased assets. Well-being in wave 4 was categorized into poor well-being (for the 1 st and 2nd tertile) and good well-being (for the 3rd and 4th tertile).

### Moderator

This study included various living arrangements as moderators to take account of the effects of different living arrangements. Unlike previous studies that primarily focused on living with or without adult children [37], this research also examined living with or without adult daughters [65], family/nonfamily members [66, 67], and adult family/nonfamily members. Each type of living arrangement was included in a model separately. Adults were defined as individuals aged 15–59, based on prior research suggesting that only adults, such as adult children, are likely to provide support to older parents [29, 68]. Adult children and daughters are individuals 15 or above. The term “children” refers to biological children, as they are typically the primary source of support for older parents in Indonesia [68]. Living with a spouse was not assessed specifically to avoid spouses at age 60 or above serving as caregivers.

### Statistical analysis

Ordered logistic regressions were used in bivariate analyses between the predictors and well-being in wave 5 and multivariable regressions. It naturally orders the well-being in wave 5 from the lowest (1st quartile) to the highest (4th quartile). Hierarchical regressions were applied to account for the effects of the covariates on the association between IADL limitations in wave 4 or changes in IADL over time and well-being in wave 5. The odds ratio (OR), 95% confidence interval (CI), and p-value < 0.05 determined the effect size, range of the effects of predictors on well-being in wave 5, and their statistical significance, respectively. The unadjusted odds ratio (UOR) refers to a model not adjusted for other covariates. The adjusted odds ratio (AOR) is for a model adjusted for other covariates.

Moderation analyses were conducted to identify whether the living arrangements and other covariates moderated the association. A p-value < 0.05 of the likelihood ratio test (LR test) means that a model with an interaction between a covariate and IADL limitations or changes in IADL limitations statistically fits better than a model without the interaction [69]. The main effects were not included in the models with the interactions, since the interactions were the focus [70, 71]. Thus, the OR showed the effect size of the interaction terms compared to the reference group.

We also assessed whether the models met key assumptions, including the proportional odds assumption, absence of multicollinearity, and the goodness-of-fit. Since p-values of chi-square for the predictors were > 0.05, the models fulfilled the odds proportional assumption. The Variance Inflation Factor (VIF) values of the predictors were lower than 5, indicating that there was no multicollinearity between predictors. Values of Akaike Information Criterion (AIC) and Bayesian Information Criterion (BIC) were yielded by comparing the models without interactions and with interactions. The models without interactions yielded lower AIC and BIC values, indicating a better fit to the data. These results suggest that the interaction terms have no significant effects on well-being.

The models did not use the longitudinal and cross-sectional weights provided by IFLS. The longitudinal weight was designed to represent the Indonesian population in 1993 and to support panel analyses, under the assumption that households and individuals included in subsequent waves were present in wave 1 [72]. The cross-sectional weight, in contrast, was constructed for cross-sectional analyses and adjusted to reflect the population size at the time of each survey. Since our sample consists of individuals living in the same households in both wave 4 and wave 5, neither the longitudinal nor the cross-sectional weights are suitable for our study design. Nevertheless, as a sensitivity analysis, we found that models estimated with person-level cross-sectional weight produced results that were consistent in both direction and magnitude with those estimated without weight.

## Results

### Descriptives of the sample

**Fig. 3:**
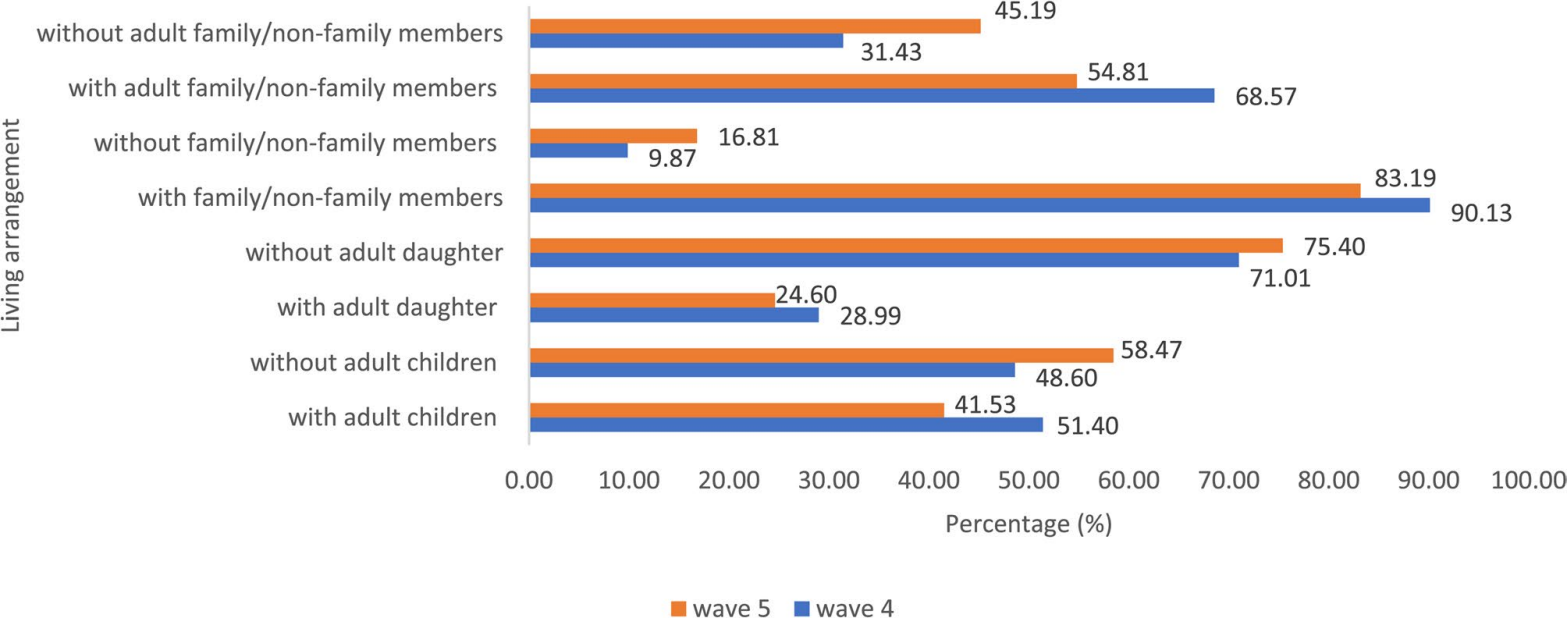
The percentages of participants based on living arrangements in wave 4 and 5 (%, *N* = 821)

Table 1 showed that in wave 4, the majority of participants were male, aged 60–69, had no IADL limitations, perceived the 1 st or 2nd quartile of well-being, and lived with family or non-family members. The mean age and standard deviation were 65.64 and 4.89, respectively. Fortunately, the percentage of those in the two lowest well-being decreased (36.54% and 31.18% in the 1 st and 2nd quartiles, respectively) in wave 5. In comparison, the two highest well-being increased (7.55% and 24.73% in the 3rd and 4th quartiles, respectively) in wave 5. Figure 3 further illustrates that living with family or non-family members remained the most common living arrangement in both waves 4 and 5. However, compared to wave 4, the percentages of those living with adult children, adult daughters, family/non-family members, or adult family/non-family members decreased in wave 5.

**Table 1:** The associations between sociodemographic characteristics in wave 4, changes across waves, and well-being in wave 5 (N=821)

Characteristics	Total		Well-being in wave 5				OR *(p*-value, 95% CI)
	**n**	**%**	^1st^	^2nd^	^3rd^	^4th^	
			**%**	**%**	**%**	**%**	
IADL limitation							
having no IADL limitations (ref)	775	94.4	35.35	31.74	7.48	25.42	0.45 (0.006, 0.25 0.80)
having any IADL limitations	46	5.6	56.52	21.74	8.70	13.04	
Changes in IADL limitation							
experiencing an improvement or the same IADL status (ref)	660	80.39	33.18	32.58	8.18	26.06	0.54 (<0.001, 0.39 0.75)
worsening IADL limitations (from having no to having any IADL limitations)	161	19.61	50.31	25.47	4.97	19.25	
Well-being index							
1^st^ quartile (ref)	206	25.09	52.91	27.67	6.80	12.62	1.59 (<0.001, 1.37 1.84)
2^nd^ quartile	366	44.58	33.61	32.51	7.10	26.78	
3^rd^ quartile	186	22.66	29.57	32.80	10.22	27.42	
4^th^ quartile	63	7.67	20.63	30.16	4.76	44.44	
Age (years old)							
60-69 (ref)	677	82.46	36.63	32.64	6.35	24.37	1.16 (0.375, 0.84 1.61)
≥ 70	144	17.54	36.11	24.31	13.19	26.39	
Gender							
female (ref)	310	37.76	40.97	30.32	8.71	20.00	1.37 (0.017, 1.06 1.77)
male	511	62.24	33.86	31.70	6.85	27.59	
Educational attainment							
never in school or not complete elementary school (ref)	475	57.86	41.05	29.68	7.16	22.11	1.23 (<0.001, 1.11 1.36)
elementary school	187	22.78	33.69	30.48	10.70	25.13	
junior high school	43	5.24	44.19	27.91	4.65	23.26	
senior high school	71	8.65	19.72	45.07	5.63	29.58	
diploma/university	45	5.48	20.00	31.11	4.44	44.44	
Asset							
low (ref)	256	31.18	41.80	30.08	8.59	19.53	1.17 (0.041, 1.01 1.36)
middle	252	30.69	35.71	28.57	6.75	28.97	
high	313	38.12	32.91	34.19	7.35	25.56	
Changes in asset							
decreased (ref)	290	35.32	38.97	35.17	6.21	19.66	1.15 (0.103, 0.97 1.36)
same	362	44.09	34.81	28.45	8.56	28.18	
increased	169	20.58	36.09	30.18	7.69	26.04	
Living with or without adult children							
with adult children at any point (ref)	479	58.34	36.53	32.15	7.93	23.38	1.07 (0.620, 0.83 1.37)
without adult children at both waves 4 & 5	342	41.66	36.55	29.82	7.02	26.61	
Living with or without adult daughters							
with adult daughters at any point (ref)	294	35.81	36.05	31.29	8.50	24.15	0.99 (0.911, 0.76 1.28)
without adult daughters at both waves 4 & 5	527	64.19	36.81	31.12	7.02	25.05	
Living with or without family or nonfamily members							
with family/non-family members at both waves 4 & 5 (ref)	664	80.88	35.24	31.33	7.98	25.45	0.76 (0.093, 0.55 1.05)
without family/non-family members at any point	157	19.12	42.04	30.57	5.73	21.66	
Living with or without adult family/non-family members							
with adult family/non-family members at any point (ref)	619	75.40	36.51	31.34	7.75	24.39	1.02 (0.900, 0.76 1.36)
without adult family/non-family members at both waves 4 & 5	202	24.60	36.54	31.18	7.55	24.73

Compared to wave 4, the percentage of older persons with no IADL limitations in wave 5 was lower. It dropped from 94.4% in wave 4 to 77.83% in wave 5. In contrast, the percentage of those with one limitation increased from 3.53% in wave 4 to 14.62% in wave 5. Similarly, the percentage of those with two limitations rose from 1.58% to 5.12%, and those with three limitations increased from 0.49% to 2.44%. Among the total participants, the percentage of older adults with limitations in shopping increased from 4.87% in wave 4 to 11.57% in wave 5. Limitations in preparing meals rose from 2.44% to 13.89%, while limitations in taking medicine increased from 0.85% to 6.70%. The figures indicated overall increases in the number of IADL limitations and each specific limitation, correlating with advancing age.

The functional capacity changed over time. Among the observations that improved from having any IADL limitations to having none, the majority (60%) had one limitation in wave 4, followed by those with two limitations (28%) and three limitations (12%) in wave 4. Conversely, among those whose functional capacity worsened from having no IADL limitations to having any, the majority (67.08%) had one limitation in wave 5, followed by those with two limitations (22.36%) and three limitations (10.56%).

The bivariate analyses in Table 1 showed that several characteristics in wave 4, such as IADL limitation, well-being, gender, educational attainment, assets, and changes in IADL limitations, had significant associations with well-being in wave 5. Older persons with any IADL limitations in wave 4 or experiencing worsening IADL limitations were less likely to perceive good well-being in wave 5. Conversely, male, higher educational attainment, more assets, and good well-being in wave 4 were positively associated with good well-being in wave 5.

### Multivariable analyses

Adjusted Model 3 in Table 2 showed that having at least one IADL limitation in wave 4 (AOR 0.41, p-value 0.003), worsening IADL limitations (AOR 0.50, *p*-value < 0.001), and decreased assets over time (AOR 0.62, *p*-value 0.003) had significant associations with poorer well-being in wave 5. Conversely, good well-being (AOR 1.50, p-value 0.005) and middle/high assets in wave 4 (AOR 1.67, *p*-value 0.003) had significant positive associations with good well-being in wave 5. Older adults with any IADL limitations were 0.41 times, those with worsening IADL limitations were 0.50 times, and those with decreased assets were less likely to perceive good well-being than those with no IADL limitations, improved/the same IADL status, and the same/increased assets, respectively. Older adults perceiving good well-being in wave 4 were 1.50 times, and those with middle/high assets were 1.67 times more likely to perceive good well-being in wave 5, compared to those perceiving poor well-being and with low assets, respectively.

**Table 2:** The associations between sociodemographic characteristics in wave 4, the changes across waves, and well-being in wave 5

Characteristics	Model 1	Model 2	Model 3
	UOR *(p*-value, 95% CI)	AOR *(p*-value, 95% CI)	AOR (*p*-value, 95% CI)
IADL limitation in wave 4 (ref: having no IADL limitations)			
having any IADL limitations	0.39(0.002, 0.22 0.70)	0.42(0.003, 0.23 0.75)	0.41 (0.003, 0.23 0.75)
Changes in IADL limitation (ref: experiencing an improvement or the same IADL status)			
worsening IADL limitations	0.51(<0.001, 0.36 0.70)	0.52(<0.001, 0.38 0.73)	0.50 (<0.001, 0.36 0.70)
Well-being at wave 4 (ref: poor well-being)			
good well-being		1.66(<0.001, 1.27 2.19)	1.50 (0.005, 1.13 1.98)
Age (ref: 60-69)			
≥ 70			1.36 (0.076, 0.97 1.90)
Gender (ref: female)			
male			1.30 (0.061, 0.99 1.70)
Educational attainment (ref: never in school or not complete elementary school)			
elementary school or higher			1.10 (0.494, 0.83 1.46)
Asset in wave 4 (ref: low)			
middle/high			1.67 (0.003, 1.19 2.34)
Changes in asset (ref: same/increased)			
decreased			0.62 (0.003, 0.45 0.85)

Model 1: IADL limitations in wave 4 and the changes in IADL limitations over time as predictors. Model 2: adjusted for well-being in wave 4. Model 3: further adjusted for age, gender, education, assets at wave 4, and the changes in assets over time

Table 3 showed that the interactions between IADL limitations and living arrangements were not statistically significant (*p*-values of LR tests > 0.05). It means that living arrangements were not significant moderators. A significant interaction effect was found only between well-being and IADL limitations in wave 4, as shown by the LR test (*p*-value 0.030, chi-square 4.69). Among older adults perceiving poor well-being in wave 4, those with IADL limitations (OR 0.29, *p*-value < 0.001) had significantly lower odds of good well-being in wave 5 compared to those without IADL limitations.

**Table 3:** Effect of having any IADL limitations in wave 4 on well-being in wave 5 moderated by the sociodemographic covariates

Characteristic	OR	*p*-value	95% CI	*p*-value of LR test (chi-square) ^a, b^
Well-being at wave 4				
poor well-being (ref)	0.29	< 0.001	0.15 0.58	0.030 (4.69)
good well-being	1.93	0.294	0.57 6.56	
Age				
60–69 (ref)	0.54	0.076	0.27 1.07	0.186 (1.75)
≥ 70	0.33	0.042	0.11 0.96	
Gender				
female (ref)	0.47	0.164	0.16 1.36	0.773 (0.08)
male	0.51	0.066	0.25 1.05	
Educational attainment				
not in school/not complete elementary school (ref)	0.58	0.133	0.28 1.18	0.143 (2.15)
elementary school or higher	0.27	0.010	0.10 0.73	
Asset				
low (ref)	0.67	0.401	0.26 1.72	0.225 (1.47)
middle & high	0.55	0.140	0.25 1.22	
Change in asset				
same & increased (ref)	0.69	0.339	0.32 1.49	0.062 (3.48)
decreased	0.15	<0.001	0.06 0.37	
Living arrangement				
with adult children at any point (ref)	0.41	0.014	0.20 0.84	0.846 (0.04)
without adult children in both waves 4 & 5	0.55	0.272	0.19 1.59	
Living arrangement				
with adult daughters at any point (ref)	0.35	0.032	1.13 0.91	0.625 (0.24)
without adult daughters in both waves 4 & 5	0.50	0.071	0.23 1.06	
Living arrangement				
with family/non-family members in both waves 4 & 5 (ref)	0.41	0.005	0.22 0.76	0.975 (0.00)
without family/non-family members at any point	0.37	0.257	0.07 2.06	
Living arrangement				
with adult family/non-family members at any point (ref)	0.44	0.010	0.23 0.82	0.852 (0.03)
without adult family/non-family members in both waves 4 & 5	0.45	0.380	0.07 2.70	

^a^Full models without interactions between predictors (well-being in wave 4, age, gender, educational attainment level, assets, changes in assets, changes in IADL limitation, and living arrangements and the changes) and IADL limitations in wave 4 were nested in models with the interactions and without the main effects (N=821). ^b^Living arrangements were only included in models with interactions between living arrangements and IADL limitation in wave 4 and without the main effects

Table 4 showed that interactions between the worsening IADL limitations and living arrangements were not statistically significant (*p*-values of LR tests > 0.05), indicating that living arrangements were not significant moderators. The figures highlighted the adverse effect of IADL limitations on well-being, regardless of co-residence type. Significant interaction between well-being in wave 4 and worsening IADL limitations was found, as indicated by the LR test (*p*-value 0,026, chi-square 4.99). Among older persons perceiving poor well-being in wave 4, those with worsening IADL limitations had lower odds of good well-being in wave 5 compared to those with improved/the same IADL status.

**Table 4:** Effect of worsening IADL limitations over time on well-being in wave 5 moderated by sociodemographic covariates

Characteristic	OR	*p*-value	95% CI	*p*-value of LR test (chi-square)^a,^^b^
Well-being at wave 4				
poor well-being (ref)	0.39	<0.001	0.26 0.59	0.026 (4.99)
good well-being	1.19	0.574	0.65 2.17	
Age				
60-69 (ref)	0.44	<0.001	0.30 0.65	0.195 (1.68)
≥ 70	0.88	0.681	0.49 1.60	
Gender				
female (ref)	0.55	0.040	0.31 0.97	0.686 (0.16)
male	0.63	0.036	0.41 0.97	
Educational attainment				
not in school/not complete elementary school (ref)	0.41	0.003	0.23 0.75	0.917 (0.01)
elementary school or higher	0.56	0.041	0.32 0.98	
Asset				
low (ref)	0.55	0.049	0.30 1.00	0.715 (0.13)
middle & high	0.82	0.415	0.51 1.32	
Change in asset				
same & increased (ref)	0.61	0.018	0.40 0.92	0.715 (0.13)
decreased	0.24	<0.001	0.13 0.43	
Living arrangement				
with adult children at any point (ref)	0.61	0.021	0.40 0.93	0.155 (2.03)
without adult children in both waves 4 & 5	0.48	0.008	0.28 0.83	
Living arrangement				
with adult daughters at any point (ref)	0.52	0.013	0.31 0.87	0.856 (0.03)
without adult daughters in both waves 4 & 5	0.54	0.011	0.33 0.87	
Living arrangement				
with family/non-family members in both waves 4 & 5 (ref)	0.47	<0.001	0.32 0.68	0.560 (1.16)
without family/non-family members at any point	0.55	0.108	0.27 1.14	
Living arrangement				
with adult family/non-family members at any point (ref)	0.52	0.001	0.35 0.76	0.446 (1.61)
without adult family/non-family members in both waves 4 & 5	0.56	0.074	0.30 1.06	

^a^Full models without interactions between predictors (well-being in wave 4, age, gender, educational attainment level, assets, changes in assets, IADL limitation in wave 4, and living arrangements and the changes) and changes in IADL limitations were nested in models with the interactions and without the main effects (N=821). ^b^Living arrangements were only included in models with interactions between living arrangements and changes in IADL limitation

## Discussion

The findings suggest that older adults with at least one IADL limitation perceive poorer well-being, aligned with previous research showing that IADL limitations at baseline negatively impact well-being at follow-up [73]. Furthermore, a worsening IADL status exacerbates well-being, as older adults tend to experience a decline in well-being when IADL limitations emerge. These results are consistent with a finding in Indonesia, which highlights that declining functional capacity significantly contributes to increased levels of depression among older adults [74].

This study also suggests that co-residence does not mitigate the disadvantageous effects of IADL limitations on well-being. Several key insights emerge from this analysis. First, regardless of the living arrangements, older adults may prefer doing physical activities without assistance to maintain their sense of functional capability [23] as well as self-care and self-worth [15] in daily and social activities. Second, although in a co-residential setting, they may not receive sufficient daily support from the co-residents or caregivers [35]. For instance, a previous study in Singapore showed that older parents received insufficient daily support. Their adult children, who live with both their young children and aging parents, must divide their caregiving between the two generations. Third, other sources may fill the gap of insufficient daily assistance. An empirical study found that the use of assistive devices, such as optical devices, helps older adults have fewer IADL limitations and lower depression [75]. Additionally, a systematic review showed that home-based rehabilitation program, such as physical exercise supervised by medical staff, improves their health and well-being [76].

Indonesian traditional culture may explain why living arrangements are insignificant in this study. Different ethnic groups exhibit distinct patterns: patrilineal tribes, such as the Batak and Dayak [77], typically live with a son or unmarried daughter [32], whereas matrilineal tribes, such as the Minangkabau [78], tend to co-reside with their daughters due to the likelihood of sons leaving for better opportunities [33]. Meanwhile, bilineal tribes, such as the Javanese, live with a son or daughter, or independently [32]. It is similar to reciprocal expectations in other Asian countries, such as China and Japan [79, 80], the living arrangements in Indonesia are intertwined with to whom the family inheritance will be transferred, as a means of financial support for the older adults [81]. Additionally, co-residence could be one of the practices of religious views that commands the younger generation to honor older persons and of filial piety as a thankful expression to them [41]. Thus, the living arrangement is not only about filial piety per se but also about financial matters as a means to support the older adults.

This study highlights the significance of financial adequacy and well-being at baseline. Older adults with low or decreased assets were associated with poorer well-being, indicating insufficient financial resources to cover health and a decent standard of living expenses [82]. That finding underlines the importance of public programs, such as national health insurance, in supporting financial stability and ensuring access to healthcare services for older adults.

The transfer of assets from older adults to their children, along with the importance of financial adequacy, may suggest that living with financially secure adult children enhances the well-being of older adults. However, this study underlines two notions. First, since the participants are the heads of households and their spouses, the assets primarily belong to them. Second, it remains unclear whether co-residing with financially secure adult children has a positive impact on well-being, given that the assets belong to the older adults. For instance, a study in China found that older adults who earn their income tend to report higher levels of well-being [83].

It was found that well-being at a younger age influences well-being in later life. That finding underscores the importance of maintaining functional capacity and financial stability during younger years to support good well-being in older age. Furthermore, the moderation analysis found that those with IADL limitations and good well-being at baseline did not experience poor well-being at follow-up. Two reasons may explain this finding. First, previous studies suggest that well-being plays a crucial role in emotional regulation [14] and self-awareness, enabling individuals to seek assistance when facing challenges in functional decline and life stressors [84]. Second, research indicates that the effects of well-being in earlier life tend to persist over time [63], suggesting that good well-being in previous years can have long-term benefits across the life course.

Our study proposes two key policy implications related to economic support and functional capacity. First, providing financial assistance to older adults with low or declining economic status is essential to help cover healthcare expenses—including assistive devices and physical rehabilitation programs—and daily living expenses. Second, raising public awareness about the importance of maintaining both functional capacity and financial health from a younger age is crucial for promoting well-being in later life.

This study has several strengths. First, as a longitudinal cohort study, it examines the effects of changes in health and living arrangements on well-being, an area not thoroughly explored in previous cross-sectional studies on IADL limitations [21, 85] or living arrangements [86]. Second, it identifies the moderating effects of living arrangements and other covariates on the relationship between functional decline, especially IADL limitations, and well-being [87]. Third, the moderation analyses reveal an insignificant effect of various living arrangements in mitigating the adverse effect of IADL limitations on well-being, which indicates the robustness of the effect of living arrangements. Fourth, this research shows that a decline in economic status worsens well-being at older age, underlining the effect of a shift in socioeconomic status to a lower level over the life course [88].

Nonetheless, this study has potential limitations. First, the smaller sample size relative to the total wave 4 or 5 sample and across subgroups may reduce statistical power, increasing the risk of Type I errors (incorrectly rejecting a true null hypothesis) and Type II errors (failing to reject a false null hypothesis) [89]. For example, IADL limitations appear to significantly reduce well-being in some subgroups when no true effect exists (Type I error), or a real moderating effect of living arrangements may be undetected (Type II error). Additionally, the sample consists of more older males than females, which may introduce selection bias and limit the generalizability of the findings to the entire older adult population in Indonesia. However, since the moderation analysis found no significant gender effect on the association of IADL limitations and well-being, this bias may be minimal.

Second, potential information bias may arise because the survey did not require medical verification of IADL limitations, leading to misclassification errors that could either exaggerate or underestimate the true relationship between IADL limitations and well-being. Third, there might be a reverse causality of the effect of poor well-being on IADL limitations. For example, depressive symptoms may induce biological changes in the body, leading to pain and impairment in physical functions [90]. Finally, unmeasured confounders may influence the results. For instance, the availability of daily physical assistance, provision of financial support, and emotional support from co-residing adult children or other individuals for older persons with IADL limitations was not assessed in the questionnaire. Moreover, sociodemographic characteristics of the co-residing adult children were not considered. Future research should incorporate these variables to have a more comprehensive understanding of the role of household support in moderating the effect of functional decline on well-being.

## Conclusions

Living with children does not mitigate the adverse effects of IADL limitations on the well-being of older adults. Meanwhile, older adults with IADL limitations or low economic status experience poorer well-being. To support this vulnerable group, policymakers should incorporate them into social service programs to enhance their well-being. There are two social programs proposed: (1) providing programs that enhance financial security and support the functional capacity among older adults, for example, free in-home exercise services, access to rehabilitation centers, and provision of assistive devices, which could improve their well-being; and (2) promoting functional capacity and financial health among younger adults for long-term well-being.

## Data Availability

The original IFLS data are available at https://www.rand.org/well-being/social-and-behavioral-policy/data/FLS/IFLS.html.

## Abbreviations

AIC: Akaike information criterion
AOR: Adjusted odds ratio
BIC: Bayesian information criterion
IADL: Instrumental activities of daily living
CES-D: Center for epidemiological studies of depression
CI: Confidence intervals
DAG: Directed acyclic graph
HRS: Health and retirement study
IFLS: Indonesian family life survey
OR: Odds ratio
LR test: Likelihood ratio test
PCA: Principal component analysis
UOR: Unadjusted odds ratio
VIF: Variance inflation factor

## References

[1] Stiglitz J, Sen A, Fitoussi J. The measurement of economic performance and social progress revisited. The measurement of economic performance and social progress revisited. Commission on the Measurement of Economic Performance and Social Progress. Paris; 2009.

[2] Liu S, Zhang W, Wu L-h, Wu B. Contributory behaviors and life satisfaction among Chinese older adults: exploring variations by gender and living arrangements. Soc Sci Med. 2019;229:70–8. 29954629 10.1016/j.socscimed.2018.06.015

[3] Srivastava S, Shaw S, Chaurasia H, Purkayastha N, Muhammad T. Feeling about living arrangements and associated health outcomes among older adults in india: a cross-sectional study. BMC Public Health. 2021;21(1):1322. 34225690 10.1186/s12889-021-11342-2 PMC8258997

[4] Hsieh N, Waite L, Disability. Psychological Well-Being, and social interaction in later life in China. Res Aging. 2019;41(4):362–89. 30636536 10.1177/0164027518824049

[5] Hsieh N, Zhang Z. Childlessness and social support in old age in China. J Cross Cult Gerontol. 2021;36(2):121–37. 33683554 10.1007/s10823-021-09427-x PMC8906474

[6] Solar OIA. A conceptual framework for action on the social determinants of health. Social Determinants of Health Discussion Paper 2 (Policy and Practice). Geneva, Switzerland: World Health Organization. 2010. https://www.who.int/publications/i/item/9789241500852. Accessed 22 December 2021.

[7] Statistics Indonesia. Indeks Kebahagiaan [Happiness Index] 2021. 2021. https://www.bps.go.id/id/publication/2021/12/27/ba1b0f03770569b5ac3ef58e/indeks-kebahagiaan-2021.html. Accessed 30 May 2024.

[8] Government of the Republic of Indonesia. Law Number 13 Year 1998 regarding Welfare of Older Persons. 1998. http://www.bphn.go.id/data/documents/98uu013.pdf. Accessed 18 December 2021.

[9] Statistics Indonesia. Statistik Penduduk Lanjut Usia [Statistics of Older Persons] 2010. 2011. https://www.bps.go.id/id/publication/2012/03/30/907d89cf92ff0ac1673d69cb/statistik-penduduk-lanjut-usia-2010.html

[10] Statistics Indonesia. Statistik Penduduk Lanjut Usia [Statistics of Older Persons] 2024. 2024. https://www.bps.go.id/id/publication/2024/12/31/a00d4477490caaf0716b711d/statistik-penduduk-lanjut-usia-2024.html

[11] Statistics Indonesia. Statistik Penduduk Lanjut Usia [Statistics of Older Persons] 2023. 2023. https://www.bps.go.id/id/publication/2023/12/29/5d308763ac29278dd5860fad/statistik-penduduk-lanjut-usia-2023.html

[12] Dhole AR, Petkar P, Choudhari SG, Mendhe H. Understanding the factors contributing to suicide among the geriatric population: A narrative review. Cureus. 2023;15(10):e46387. 37927668 10.7759/cureus.46387 PMC10620465

[13] VanderWeele TJ, Trudel-Fitzgerald C, Allin P, Farrelly C, Fletcher G, Frederick DE, Hall J, Helliwell JF, Kim ES, Lauinger WA, et al. Current recommendations on the selection of measures for well-being. Prev Med. 2020;133:106004. 32006530 10.1016/j.ypmed.2020.106004

[14] Delle Fave A, Bassi M, Boccaletti ES, Roncaglione C, Bernardelli G, Mari D. Promoting Well-Being in old age: the psychological benefits of two training programs of adapted physical activity. Front Psychol. 2018;9:828. 29910755 10.3389/fpsyg.2018.00828 PMC5992429

[15] Saadeh M, Welmer A-K, Dekhtyar S, Fratiglioni L, Calderón-Larrañaga A. The role of psychological and social Well-being on physical function trajectories in older adults. Journals Gerontology: Ser A. 2020;75(8):1579–85. 10.1093/gerona/glaa114 PMC7357580 32384140

[16] Ryan RM, Deci EL. Self-determination theory and the facilitation of intrinsic motivation, social development, and well-being. Am Psychol. 2000;55(1):68–78. 11392867 10.1037//0003-066x.55.1.68

[17] Mlinac ME, Feng MC. Assessment of activities of daily Living, Self-Care, and independence. Arch Clin Neuropsychol. 2016;31(6):506–16. 27475282 10.1093/arclin/acw049

[18] Ando T, Nishimoto Y, Hirata T, Abe Y, Takayama M, Maeno T, Fujishima S, Takebayashi T, Arai Y. Association between multimorbidity, self-rated health and life satisfaction among independent, community-dwelling very old persons in japan: longitudinal cohort analysis from the Kawasaki ageing and Well-being project. BMJ Open. 2022;12(2):e049262. 35210335 10.1136/bmjopen-2021-049262 PMC8883229

[19] Kiyoshige E, Kabayama M, Gondo Y, Masui Y, Inagaki H, Ogawa M, Nakagawa T, Yasumoto S, Akasaka H, Sugimoto K, et al. Age group differences in association between IADL decline and depressive symptoms in community-dwelling elderly. BMC Geriatr. 2019;19(1):309. 31722665 10.1186/s12877-019-1333-6 PMC6854629

[20] Handajani Y, Schröder-Butterfill E, Hogervorst E, Turana Y, Hengky A. Depression among older adults in indonesia: Prevalence, role of chronic conditions and other associated factors. Clin Pract Epidemiol Mental Health. 2022;18. 10.2174/17450179-v18-e2207010 PMC10156049 37274861

[21] Chen T. Living arrangement preferences and realities for elderly chinese: implications for subjective wellbeing. Aging Soc. 2019;39(8):1557–81.

[22] Menec VH. The relation between everyday activities and successful aging: A 6-Year longitudinal study. Journals Gerontology: Ser B. 2003;58(2):S74–82. 10.1093/geronb/58.2.s74 12646596

[23] Wróblewska Z, Chmielewski JP, Florek-Łuszczki M, Nowak-Starz G, Wojciechowska M, Wróblewska IM. Assessment of functional capacity of the elderly. Ann Agric Environ Med. 2023;30(1):156–63. 36999869 10.26444/aaem/161775

[24] da Silva TBL, Dos Santos G, Moreira APB, Ishibashi GA, Verga CER, de Moraes LC, Lessa PP, Cardoso NP, Ordonez TN, Brucki SMD. Cognitive interventions in mature and older adults, benefits for psychological well-being and quality of life: a systematic review study. Dement Neuropsychol. 2021;15(4):428–39. 35509795 10.1590/1980-57642021dn15-040002 PMC9018088

[25] Gates N, Valenzuela M, Sachdev PS, Singh MA. Psychological well-being in individuals with mild cognitive impairment. Clin Interv Aging. 2014;9:779–92. 24855347 10.2147/CIA.S58866 PMC4020883

[26] Fujiwara Y, Shinkai S, Kumagai S, Amano H, Yoshida Y, Yoshida H, Kim H, Suzuki T, Ishizaki T, Haga H, et al. Longitudinal changes in higher-level functional capacity of an older population living in a Japanese urban community. Arch Gerontol Geriatr. 2003;36(2):141–53. 12849088 10.1016/s0167-4943(02)00081-x

[27] Murakami K, Tsubota-Utsugi M, Satoh M, Asayama K, Inoue R, Ishiguro A, Matsuda A, Kanno A, Yasui D, Murakami T, et al. Impaired Higher-Level functional capacity as a predictor of stroke in Community-Dwelling older adults. Stroke. 2016;47(2):323–8. 26732573 10.1161/STROKEAHA.115.011131

[28] Kahana E, Lovegreen L, Kahana B, Kahana M. Person, Environment, and Person-Environment fit as influences on residential satisfaction of elders. Environ Behav. 2003;35(3):434–53.

[29] Johar M, Maruyama S. Intergenerational cohabitation in modern indonesia: filial support and dependence. 2011;20(S1):87–104. 10.1002/hec.1708 21274997

[30] Cameron L. The residency decision of elderly indonesians: A nested logit analysis. Demography. 2000;37(1):17–27. 10748986

[31] Priebe J. Old-age poverty in indonesia: measurement issues and living arrangements. 2017; 48(6):1362–85.

[32] Beard VA, Kunharibowo Y. Living arrangements and support relationships among elderly indonesians: case studies from Java and Sumatra. Int J Popul Geogr. 2001;7(1):17–33.

[33] Kreager P, Schröder-Butterfill E. Indonesia against the trend? Ageing and inter-generational wealth flows in two Indonesian communities. Demographic Res. 2008;19(52):1781–810. 10.4054/DemRes.2008.19.52 PMC3672954 23750113

[34] Hogervorst E, Schröder-Butterfill E, Handajani YS, Kreager P, Rahardjo TBW. Dementia and dependency vs. Proxy indicators of the active ageing index in Indonesia. Int J Environ Res Public Health. 2021;18(16). 10.3390/ijerph18168235 PMC8391311 34443985

[35] Tan K-K, He H-G, Chan SW-C, Vehviläinen-Julkunen K. The experience of older people living independently in Singapore. Int Nurs Rev. 2015;62(4):525–35. 26058716 10.1111/inr.12200

[36] Teerawichitchainan B, Knodel J, Pothisiri W. What does living alone really mean for older persons? A comparative study of Myanmar, Vietnam, and Thailand. Demographic Res. 2015;S15(48):1329–60.

[37] Roystonn K, Abdin E, Shahwan S, Zhang Y, Sambasivam R, Vaingankar JA, Mahendran R, Chua HC, Chong SA, Subramaniam M. Living arrangements and cognitive abilities of community-dwelling older adults in Singapore. Psychogeriatrics. 2020;20(5):625–35. 32141156 10.1111/psyg.12532

[38] Lakey B, Cohen S. Social support theory and measurement. In: Cohen S, Underwood LG, Gottlieb BH, editors. Social support measurement and intervention: A guide for health and social scientists. New York, US: Oxford University Press; 2000. pp. 29–52.

[39] Dommaraju P. One-person households in India. Demographic Res. 2015;S15(45):1239–66.

[40] Cheung C-k, Kwan AY-h, Ng SH. Impacts of filial piety on preference for kinship versus public care. J Community Psychol. 2006;34(5):617–34.

[41] Wianto E, Sarvia E, Chen C-H. Authoritative parents and dominant children as the center of communication for sustainable healthy aging. Int J Environ Res Public Health. 2021;18(6):3290. 33810112 10.3390/ijerph18063290 PMC8004678

[42] Teerawichitchainan B, Pothisiri W, Long GT. How do living arrangements and intergenerational support matter for psychological health of elderly parents? Evidence from Myanmar, Vietnam, and Thailand. Soc Sci Med. 2015;136–137:106–16. 25993521 10.1016/j.socscimed.2015.05.019

[43] Suvanno S, Otakum N, Utapao K, Chuanwan S, Ruangsan N. Perception, attitude and Preparation for aged society in Thailand among people in Bangkok. J Pharm Negat Results. 2022;617–23.

[44] Bai X, Lai DWL, Liu C. Personal care expectations: Photovoices of Chinese ageing adults in Hong Kong. Health & Social Care in the Community. 2020;28(3):1071–81. 31919932 10.1111/hsc.12940 PMC7187378

[45] Gubhaju B, ØStbye T, Chan A. Living arrangements of community-dwelling older singaporeans: predictors and consequences. Aging Soc. 2018;38(6):1174–98.

[46] Setiadi S, Hidayah S. Subjective Well-Being amongst older women from migrant and Non-Migrant households in rural Java, Indonesia. J Popul Social Stud (JPSS). 2021;29:459–78.

[47] Hernán MA, Robins JM. Causal inference: what if. 1st ed. Boca Raton: Chapman & Hall/CRC; 2020.

[48] Gautam R, Saito T, Kai I. Leisure and religious activity participation and mental health: gender analysis of older adults in Nepal. BMC Public Health. 2007;7(1):299. 17953749 10.1186/1471-2458-7-299 PMC2140059

[49] Sereny M. Living arrangements of older adults in china: the interplay among Preferences, Realities, and health. Res Aging. 2011;33(2):172–204.

[50] RAND Corporation. RAND Family Life Survey. https://www.rand.org/well-being/social-and-behavioral-policy/data/FLS.html

[51] Strauss J, Witoelar F, Sikoki B. The fifth wave of the Indonesia family life survey: overview and field report. Volume 1. Santa Monica, CA: RAND Corporation; 2016.

[52] Spiers GF, Liddle JE, Stow D, Searle B, Whitehead IO, Kingston A, Moffatt S, Matthews FE, Hanratty B. Measuring older people’s socioeconomic position: a scoping review of studies of self-rated health, health service and social care use. J Epidemiol Community Health. 2022;76(6):572–9. 35292509 10.1136/jech-2021-218265 PMC9118079

[53] Strauss J, Witoelar F, Sikoki B, Wattie AM. The fourth wave of the Indonesia family life survey (IFLS4. Overview and field report. Volume 1. Santa Monica, CA: RAND Corporation; 2009. https://www.rand.org/well-being/social-and-behavioral-policy/data/FLS/IFLS.html.

[54] Klingstedt ML, Wångby-Lundh M, Olsson T, Ferrer-Wreder L. Reliability and construct validity of five life domains in the adolescent drug abuse diagnosis instrument in a sample of Swedish adolescent girls in special residential care. Nordisk Alkohol Nark. 2020;37(4):411–26. 35310922 10.1177/1455072520941990 PMC8899243

[55] Santos RO, Gorgulho BM, Castro MA, Fisberg RM, Marchioni DM, Baltar VT. Principal component analysis and factor analysis: differences and similarities in nutritional epidemiology application. Rev Bras Epidemiol. 2019;22:e190041. 31365598 10.1590/1980-549720190041

[56] Björgvinsson T, Kertz SJ, Bigda-Peyton JS, McCoy KL, Aderka IM. Psychometric properties of the CES-D-10 in a psychiatric sample. Assessment. 2013;20(4):429–36. 23513010 10.1177/1073191113481998

[57] Taber KS. The use of cronbach’s alpha when developing and reporting research instruments in science education. Res Sci Educ. 2018;48(6):1273–96.

[58] Zheng W, Huang Z. Onset of ADL and IADL limitation among Chinese middle-aged and older adults. PLoS ONE. 2023;18(7):e0287856. 37459324 10.1371/journal.pone.0287856 PMC10351716

[59] Ćwirlej-Sozańska A, Wiśniowska-Szurlej A, Wilmowska-Pietruszyńska A, Sozański B. Determinants of ADL and IADL disability in older adults in southeastern Poland. BMC Geriatr. 2019;19(1):297. 31672121 10.1186/s12877-019-1319-4 PMC6824102

[60] Chalise HN, Saito T, Kai I. Functional disability in activities of daily living and instrumental activities of daily living among Nepalese newar elderly. Public Health. 2008;122(4):394–6. 17888469 10.1016/j.puhe.2007.07.015

[61] Arokiasamy P, Uttamacharya U, Jain K, Biritwum RB, Yawson AE, Wu F, Guo Y, Maximova T, Espinoza BM, Salinas Rodríguez A, et al. The impact of Multimorbidity on adult physical and mental health in low- and middle-income countries: what does the study on global ageing and adult health (SAGE) reveal? BMC Med. 2015;13(1):178. 26239481 10.1186/s12916-015-0402-8 PMC4524360

[62] Feng Z. Childlessness and vulnerability of older people in China. Age Ageing. 2017;47(2):275–81. 10.1093/ageing/afx137 PMC6016684 29096004

[63] Chen Y, Sun R. The impact of children’s gender on parent’s mental health and cognition -- evidence from China. SSM - Popul Health. 2022;18:101086. 35464614 10.1016/j.ssmph.2022.101086 PMC9019397

[64] Howe LD, Galobardes B, Matijasevich A, Gordon D, Johnston D, Onwujekwe O, Patel R, Webb EA, Lawlor DA, Hargreaves JR. Measuring socio-economic position for epidemiological studies in low- and middle-income countries: a methods of measurement in epidemiology paper. Int J Epidemiol. 2012;41(3):871–86. 22438428 10.1093/ije/dys037 PMC3396323

[65] Chen F, Short SE. Household context and subjective Well-Being among the oldest old in China. J Fam Issues. 2008;29(10):1379–403. 19554216 10.1177/0192513X07313602 PMC2701306

[66] Agrawal S, EFFECT OF LIVING ARRANGEMENT ON, THE HEALTH STATUS OF ELDERLY IN INDIA. Asian Popul Stud. 2012;8(1):87–101. 28868080 10.1080/17441730.2012.646842 PMC5575814

[67] Banjare P, Dwivedi R, Pradhan J. Factors associated with the life satisfaction amongst the rural elderly in Odisha, India. Health Qual Life Outcomes. 2015;13(1):201. 26691176 10.1186/s12955-015-0398-y PMC4687085

[68] Witoelar F. Household Dynamics and Living Arrangements of the Elderly in Indonesia: Evidence from a Longitudinal Survey. In: Aging in Asia: Findings from New and Emerging Data Initiatives. Washington, DC: The National Academies Press. 2012. https://nap.nationalacademies.org/read/13361/chapter/14. Accessed 22 June 2021.

[69] Beck CW, Bliwise NG. Interactions are critical. CBE Life Sci Educ. 2014;13(3):371–2. 25185220 10.1187/cbe.14-05-0086 PMC4152198

[70] STATA UCLA. What happens if you omit the main effect in a regression model with an interaction? | Stata FAQ [https://stats.oarc.ucla.edu/stata/faq/what-happens-if-you-omit-the-main-effect-in-a-regression-model-with-an-interaction/

[71] Knol MJ, VanderWeele TJ. Recommendations for presenting analyses of effect modification and interaction. Int J Epidemiol. 2012;41(2):514–20. 22253321 10.1093/ije/dyr218 PMC3324457

[72] Strauss J, Witoelar F, Sikoki B, Wattie AM. User’s Guide for the Indonesia Family Life Survey, Wave 4 Volume 2. Santa Monica, CA. 2009.

[73] Liu Y, Yang X, Xu Y, Wu Y, Zhong Y, Yang S. Cognitive function and depressive symptoms among Chinese adults aged 40 years and above: the mediating roles of IADL disability and life satisfaction. Int J Environ Res Public Health. 2023;20(5):4445. 36901451 10.3390/ijerph20054445 PMC10002125

[74] Gunawan I, Lin M-H, Hsu H-C. Exploring the quality of life and its related factors among the elderly. South East Asia Nurs Res. 2020;2:1.

[75] Horowitz A, Brennan M, Reinhardt JP, MacMillan T. The impact of assistive device use on disability and depression among older adults with Age-Related vision impairments. Journals Gerontology: Ser B. 2006;61(5):S274–80. 10.1093/geronb/61.5.s274 16960241

[76] Alves E, Gonçalves C, Oliveira H, Ribeiro R, Fonseca C. Health-related outcomes of structured home-based rehabilitation programs among older adults: A systematic literature review. Heliyon. 2024;10(15):e35351. 39170553 10.1016/j.heliyon.2024.e35351 PMC11336612

[77] Ananta A, Arifin EN, Hasbullah MS, Handayani NB, Pramono A. Demography of indonesia’s ethnicity. Singapore: ISEAS–Yusof Ishak Institute; 2014.

[78] Kunto YS, Bras H. Ethnic group differences in dietary diversity of School-Aged children in indonesia: the roles of gender and household SES. FoodNutr Bull. 2019;40(2):182–201. 10.1177/0379572119842993 31046454

[79] Deng WJ, Hoekstra JSCM, Elsinga MG. Why women own less housing assets in china? The role of intergenerational transfers. J Housing Built Environ. 2019;34(1):1–22.

[80] Izuhara M. Changing family tradition: housing choices and constraints for older people in Japan. Hous Stud. 2000;15(1):89–110.

[81] Arifin E. Living arrangements of older persons in East Java, Indonesia. Asia-Pacific Popul J. 2006;21:93–112.

[82] Abruquah LA, Yin X, Ding Y. Old age support in urban china: the role of pension schemes. Self-Support Ability Intergenerational Assistance. 2019;16(11):1918. 10.3390/ijerph16111918 PMC6603691 31151220

[83] Liu X, Wang Z, Zhang C, Zhang C, Peng L, Xu H. Effects of income on subjective Well-Being in the elderly: complete mediation roles of Self-Rated health and psychological capital. INQUIRY: J Health Care Organ Provis Financing. 2024;61:00469580241284967. 10.1177/00469580241284967 PMC11456172 39314000

[84] Rasnayake S, Navratil P. Warning signs of elderly suicide and factors affecting professional Help-Seeking: case of Sri Lanka. J Popul Social Stud [JPSS]. 2022;31:1–19.

[85] Gumà J. What influences individual perception of health? Using machine learning to disentangle self-perceived health. SSM - Popul Health. 2021;16:100996. 34917748 10.1016/j.ssmph.2021.100996 PMC8669356

[86] Yao Y, Ding G, Wang L, Jin Y, Lin J, Zhai Y, Zhang T, He F, Fan W. Risk factors for depression in empty nesters: A Cross-Sectional study in a coastal City of Zhejiang Province and China. Int J Environ Res Public Health. 2019;16(21):4106. 31653106 10.3390/ijerph16214106 PMC6862174

[87] Bauman AE, Sallis JF, Dzewaltowski DA, Owen N. Toward a better Understanding of the influences on physical activity: the role of determinants, correlates, causal variables, mediators, moderators, and confounders. Am J Prev Med. 2002;23(2 Suppl):5–14. 12133733 10.1016/s0749-3797(02)00469-5

[88] Pan T, Li C, Zhou Y. Life course socioeconomic position and care dependency in later life: a longitudinal multicohort study from 17 countries. eClinicalMedicine. 2025;79. 10.1016/j.eclinm.2024.102994 PMC11683273 39737217

[89] Knudson DV, Lindsey C. Type I and type II errors in correlations of various sample sizes. Compr Psychol. 2014;3:03.CP.03.01.

[90] Trivedi MH. The link between depression and physical symptoms. Prim Care Companion J Clin Psychiatry. 2004;6(Suppl 1):12–6. 16001092 PMC486942

